# Social networks, mobility, and HIV risk among women in the fishing communities of Lake Victoria

**DOI:** 10.1186/s12905-022-02144-8

**Published:** 2022-12-28

**Authors:** Sarah Nakamanya, Elialilia S. Okello, Zachary A. Kwena, Gertrude Nanyonjo, Ubaldo M. Bahemuka, Freddie M. Kibengo, Ali Ssetaala, Elizabeth A. Bukusi, Saidi Kapiga, Patricia E. Fast, Janet Seeley

**Affiliations:** 1 Medical Research Council/Uganda Virus Research Institute and London School of Hygiene and Tropical Medicine (MRC/UVRI and LSHTM), Uganda Research Unit, P.O. Box 49, Entebbe, Uganda; 2 Mwanza Intervention Trials Unit (MITU), National Institute for Medical Research, Mwanza, Tanzania; 3 Research Care and Training Program (RCTP), Centre for Microbiology Research, Kenya Medical Research Institute (KEMRI), Nairobi, Kenya; 4 UVRI-IAVI HIV Vaccine Program, Entebbe, Uganda; 5 International AIDS Vaccine Initiative (IAVI), New York, USA; 6 Pediatric Infectious Diseases, School of Medicine, Stanford University, Palo Alto, CA USA; 7 Department of Global Health and Development, London School of Hygiene and Tropical Medicine, London, UK

**Keywords:** Fishing communities, Social networks, Mobility, HIV risk, Women, Lake Victoria

## Abstract

**Background:**

Population mobility is a demonstrated barrier to reducing HIV incidence. A clear understanding of social networks and their influence on mobility among women in the fishing communities of Lake Victoria may contribute to tailoring effective interventions that suit the needs of these mobile women.

**Methods:**

A cross-sectional qualitative methods study was conducted to understand mobility patterns among women resident and or working in fishing communities of Lake Victoria in Kenya, Tanzania, and Uganda. The study was conducted in six fishing communities from March 2018 to June 2019. The communities were purposively selected, based on population size (1000 people or more) and HIV prevalence of > 15% among women aged 18 years or older who had lived in the fishing community for at least six months. In-depth interviews were conducted with 24 key informants and 72 women from the sites in the three countries. Questions focused on women’s social networks and other factors that fuelled or facilitated women’s mobility as well as challenges they faced due to mobility. Data analysis followed a thematic framework approach.

**Results:**

Different social groupings/networks existed among women in the fishing communities of Lake Victoria. These included female sex workers, women fish processors/traders, women bar workers/owners, restaurant workers, and family networks. Networks encouraged mobility, supporting finding work opportunities, but also increased sexual risks through partner changes. The benefits of networks included information sharing, financial support, and group protection, especially against violence.

**Conclusion:**

Social networks and groupings among women in the fishing communities of Lake Victoria could be useful in tailoring HIV prevention and HIV care interventions to suit the needs of these highly mobile populations.

**Supplementary Information:**

The online version contains supplementary material available at 10.1186/s12905-022-02144-8.

## Background

The global HIV burden remains high, with an estimated 1.5 million new infections in 2020 [1]. Research on risk factors for HIV infection has indicated that there is a relationship between mobility and HIV spread [2–8]. High levels of population mobility present a challenge to the global HIV response [5, 9], both in terms of the risk of new infections and the risk of treatment interruption [9–11]. There is also evidence that members of mobile populations are less likely to test and/or link to HIV care and treatment [12, 13]. Members of fishing communities on Lake Victoria have been shown to be highly mobile, often moving due to changes in fish seasons and in search of income [14, 15].

Over the past two decades, a higher HIV burden has been demonstrated among highly mobile populations like fishing communities; particularly among women [16–20]. Women’s mobility has been shown to increase their risk of HIV acquisition as it increases their susceptibility to involvement in unprotected sex and potentially harmful alcohol use, which result from lower paid and riskier work that women engage in, including fish processing and bar work [14], in addition to hampering the effective delivery of interventions [13, 21]. Among fishing communities on the shores of Lake Victoria, Lake Malawi, and the Kafue Flats in Zambia, an association has been shown between risk behaviour like sex-for-fish/cash relationships and mobility [22–25]. The sex-for-fish relationships in the Lake Victoria fishing communities are attributed to a combination of factors, which include the reduced fish stocks in the lake and resulting competition for fish among female fish buyers [26, 27].

Social networks play a part in influencing where people travel, with migrants often moving to places where they have family members or friends [28]. A social network is a linked group of individuals who know each other and may provide social support in, for example, accessing income opportunities and/or social benefits. These networks can also have a significant effect on involvement in risky work including bar work and sex work as well as the adoption of new behaviour [9, 29]. A clear understanding of the social networks, and how they influence mobility and HIV risk, can help inform the design of HIV prevention and care interventions that are tailored to suit the needs of mobile people in fishing communities on Lake Victoria.

In this paper, we describe different social networks, mobility patterns and risk of HIV acquisition among women resident and/or working in fishing communities in the three East African countries bordering Lake Victoria: Kenya, Tanzania and Uganda.

## Methods

### Study design and settings

This was a multi-site study conducted by the partner Institutions of the Lake Victoria Consortium for Health Research (LVCHR) at the different sites in Kenya, Uganda and Tanzania. It was a cross-sectional qualitative methods study conducted among individuals in fishing communities on the shores of Lake Victoria. In-depth interviews and participant observation were used to gather data on women’s mobility patterns among women resident and or working in fishing communities. The study was conducted in six fishing communities (two from each country) between March 2018 and June 2019. The fishing communities were purposively selected, based on a known HIV prevalence of > 15% among women and with a population of at least 1000 people in each community. We defined a fishing community as consisting of one or more landing sites on either the mainland or an island where people were focused on fishing and fishing-related activities and lived together in a defined geographical area. In some cases, fishing communities coincided with administrative boundaries such as villages and council wards. Landing sites in these fishing communities are often very busy, attracting many people who come to engage in fish-related activities, including fish buying or selling resulting in high levels of mobility. The movements were usually in the form of regular rotational movements between the different fishing communities as well as surrounding towns and rural areas, lasting days, weeks or even months. The length of travel was usually determined by the reason for moving or type of work women did [14].

The study population comprised adults (men and women) aged 18 years or older and living in the fishing community for at least six months.

### Sampling and data collection

Members of the Consortia leadership conceived and designed the study. Each site had one senior social science/behavioural researcher who coordinated activities of the study including protocol training, supervision of field teams in data collection and management activities.

**Fig. 1:**
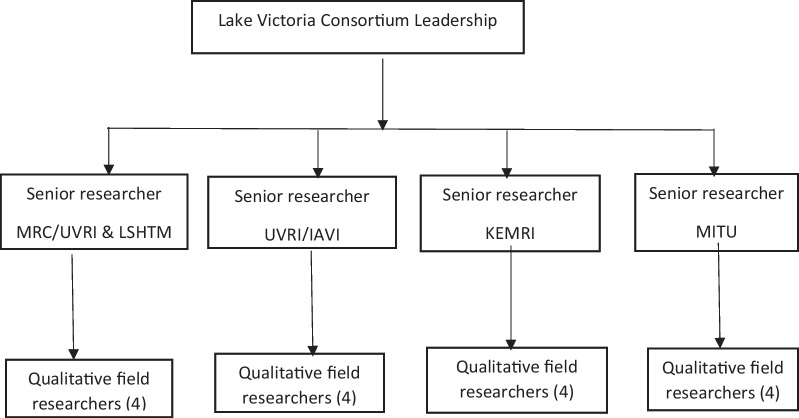
Flowchart on structure of research teams

There were four field researchers at each site, both male and female field researchers of various ages (ranging between 23 and 40), who were selected from the research teams at each of the participating institutions based on their experience and training. Figure 1 shows the composition of the research teams. Although the field researchers were not members of the social networks, they had enough experience and rapport building skills to conduct research in the study settings.

The initial stage of the study involved a community entry visit in each of the six purposively selected fishing communities to introduce the study and the research teams to the local leaders and other community gatekeepers and obtain their support and community consent. With the help of these gatekeepers, participants were identified and those who met the eligibility criteria (aged 18 years and older, lived in the fishing community for at least six months and were interested) were given detailed information about the study. Eligibility of identified participants was determined through engaging individuals in an informal set of questions about their age and length of residence in the community. Again, through our meetings with the gatekeepers, we identified information-rich categories of people who interacted a lot with the women and who we could approach for key informant interviews. These included beach management officers, elders, employers, peer leaders, village health teams (VHTs), transporters, bar/lodge managers and community local leaders. Individuals willing to participate signed an informed consent form prior to participation.

In each fishing community, data were collected using participant observation for two months, and in-depth interviews with both male and female key informants (six in each fishing community and equal numbers by gender). Eighteen interviews in each fishing community were conducted with women from three different occupational backgrounds, including women working in entertainment facilities and hotels/restaurants, women involved in fish processing or trade and women residing in the fishing community because they were, for example, cohabiting with a spouse or partner. All interviews were conducted in a convenient place within the community, either in the participant’s home, workplace, or another place that was convenient and safe with adequate privacy for both the participant and researcher. No audio recorders were used. Interviewers were trained to conduct interviews without recorders as recorders could often suppress the discussion, especially on sensitive topics [30]. Interviewers took short notes (in English) during the interview, while maintaining participants’ words in the local language as quotes to illustrate interesting information that came up during the interview. Notes were expanded, quotes were translated, and written interview scripts were produced within 24 h after the interview and before conducting another interview. Interviews were conducted using an interview guide which was developed in English and translated into the local languages of Dholuo, Kiswahili and Luganda for use, respectively, in Kenya, Tanzania and Uganda. Questions were asked about women’s lives and livelihoods, women’s mobility patterns and the gender-specific issues around mobility, including social ties as well as group mobility. On average, interviews lasted about one hour. To ensure data accuracy and completeness, debriefing meetings were held among the various teams and any gaps identified were addressed during the remaining interviews.

For participant observation, researchers participated in the life of the community at different places and times throughout the day including night-time and weekends. Observations focused on entry and exit points, meeting spots, places where women worked, landing sites, bars, and other communal areas, to determine which women moved, for which purpose, signs of group mobility, and to learn where the women were coming from and when they came to a particular place. Researchers engaged in informal conversations with community members for clarity on observed issues and would from time to time return to a private place within the community to jot down notes of their observations. Detailed notes were written up at the end of each day.

### Data management and analysis

Data analysis took a thematic framework approach and was an iterative process guided by Braun and Clarke’s thematic analytical process [31–33], whereby interview scripts and notes from participant observation from the different sites were initially read and re-read independently by the researchers to familiarize themselves with the material. The researchers independently identified initial codes, the descriptive labels for information relevant to the research question. The teams later discussed and agreed on the codes. Codes from the different data sources, including key informant interviews, interviews with the women as well as observation notes were compared for similarities. Codes with similar or close meanings were grouped together to generate broader themes. The emergent themes were shared and discussed amongst the researchers at the different sites and a thematic coding framework was agreed upon across the sites. Data were charted on a matrix under relevant/matching themes to condense it in order to enable quick and easy navigation [33]. The data sets from the different sites were merged to produce a single worksheet, with unique identifiers to differentiate data from the different sites and samples. The merged dataset allowed easy navigation through, and comparison of, the data from the different sites. The analysis was done as a team: the researchers from the different sites had calls regularly to compare analytical notes and to discuss and resolve issues arising from the data. Patterns and themes that portrayed women’s livelihoods, the different social groupings and how they operated, the benefits as well as risks within the different social groupings were also identified and these formed the basis of this paper (see Additional file 1).

## Results

### Participants' demographic characteristics

A total of 96 interviews—72 with women from different occupational backgrounds and 24 with male and female key informants, were conducted across the different sites, with numbers evenly distributed across the sites (see Table 1).

**Table 1:** Characteristics of interview participants

Variables	MRC/UVRI and LSHTM			UVRI-IAVI			MITU			KEMRI			Total
	KII		IDI	KII		IDI	KII		IDI	KII		IDI	N = 96
	Male	Female		Male	Female		Male	Female		Male	Female		
*Sex*													
Male	3	0	0	3	0	0	3	0	0	3	0	0	**12**
Female	0	3	18	0	3	18	0	3	18	0	3	18	**84**
*Age (years)*													
18–24	0	0	8	0	0	2	0	0	2	0	0	0	**12**
25–34	0	0	6	0	0	8	2	0	4	0	1	4	**25**
35+	3	3	4	3	3	8	1	3	12	3	2	14	**59**
*Education*													
None	1	0	2	0	0	0	0	1	2	*	–*	–*	**6**
Primary	1	2	8	2	2	10	1	2	13	–*	–*	–*	**41**
Secondary	1	1	7	1	1	8	2	0	3	–*	–*	–*	**24**
Tertiary	0	0	1	0	0	0	0	0	0	–*	–*	–*	**1**
*Occupation*													
Fish related activities	2	1	4	2	2	3	2	2	9	3	3	2	**35**
Trader	0	0	1	0	0	2	0	0	2	0	0	6	**11**
Sex/bar worker	1	1	8	1	1	8	0	0	3	0	0	0	**23**
Other	0	1	5	0	0	5	1	1	4	0	0	10	**27**
*Marital status*													
Married/cohabiting	3	1	8	2	2	6	1	2	9	3	3	5	**45**
Separated/single	0	1	10	1	0	12	2	1	9	0	0	7	**43**
Widowed	0	1	0	0	1	0	0	0	0	0	0	6	**8**

Bold denotes the line total

*Missing data

#### Migrating to the fishing community

When moving into the fishing community, women followed social/family connections with most reporting that they came at the invitation of a friend, relative or sexual partner. After arriving in the fishing community, most women became involved in fish processing or trading while others engaged in bar/restaurant work, market vending as well as sex work. Participants noted that in the past, there was a lot of internal migration around the different fishing communities, but some people now had more permanent places of residence within the fishing communities, which they considered as their homes. So, even when they moved, some returned to these particular places. Most of the movement rotated around work although some women reportedly moved in search of health care including ante-natal and HIV care, others moved because of family obligations and to a small extent for leisure. Detailed mobility patterns of the women are published elsewhere [14].

#### Sharing of information

Most of the movement, especially for work, was triggered by the different social connections women had, and was usually aided by mobile phone calls, phone text messages and social media, especially WhatsApp. Within the networks, members shared mobile phone contacts, which helped to cement these relationships. Mobile phones also enabled network members to stay connected and to easily get together when alerted. Due to the easy means of communication, they could quickly decide on a course of action to take. Network members always kept in touch and were kept up-to-date especially with information on where business was good, through the different communication platforms. Through the friendship networks that they created and using these channels to easily share information, particularly regarding work, across and within a network, members were able to keep their trade/dealings moving.

These women, especially bar workers and sex workers rely on information from other places whether there is money or not. So, when their counterparts on other landing sites or islands do not communicate about the situation there, they take longer to move. When they are told about what is happening in other places; that is when they can move (man, 34 years, key informant -youth leader, Uganda [UG1]).

#### Social groupings within the fishing communities

Diverse types of groupings existed within the fishing communities. These groupings or networks were formed based on several features which included similar characteristics and interests of peers/members or involvement in similar jobs. The most salient among the various groupings that existed in fishing communities were the sex workers’ network, women fish traders/processors, bar owners/workers’ network, hotel/restaurant workers’ network, sex workers linking with lodge operators and women’s ties to their family networks. However, almost all the different social groupings also networked with the fishermen, who were their main sexual clients/partners or fish suppliers. An important feature to note is that even members of other networks apart from sex workers engaged in transactional sex, especially with the fishermen. The networks were quite similar across the different sites and they also tended to follow similar practices, including sharing information, offering group protection and following fishermen.

##### Sex workers’ networks

Those who usually frequented the fishing community during fish harvesting seasons to target fishermen and other men who were interested in paying for sex were women and girls under the age of 40 years. Women selling sex had a network, which not only recruited other young girls/ women into the trade but also trained the recruits on ways of surviving in this industry including educating them about condom use, sex charges, and other forms of support.

The women also shared information about places where business was good, sometimes being provided with information by fishermen who befriended them. Their networks helped them to find employment as well as social security whereby they saved, donated and lent money to one another through their associations known as *‘Ifogong'o’* in Sukuma language (meaning *savings pouch/purse* [in Tanzania-TZ] and **‘***Munno mukabi***’** in Luganda language (meaning ‘a friend in need’ self-help group [in Uganda-UG]).

If one of them met with bad customers, she makes effort to know who he is and shares the information with others in their network (woman, bar worker, 38 years, TZ)

These groups have helped us to save and to borrow in order to meet our financial needs. When we save, you can borrow the money among yourselves. These social coordinates are useful, and they are the ones where we find comfort. They include the friends and those you associate with like your peers (woman, 31 years, bar worker, UG2).

Almost all work-related movements by sex workers were prompted by phone calls from or to fellow sex workers in other ‘lucrative’ locations. They had connections who alerted them that fishermen in a certain area were making big catches. They would then move into that area quickly, leaving when their takings diminished, or they received information of another place where work was available. For example, before moving to a specific area, they or a member of the group who could relay information to other group members, first got phone calls either from fellow sex workers or from the lodge owners/operators. The latter usually kept sex workers’ phone contacts and in addition to providing them with a place to work (to provide sex), they alerted them to come whenever the fish season started, and fishermen had money. So, in most of their movements, the women relied on this information from their networks and usually carried small notebooks where they jotted down useful information like locator information of the contact people in their respective destinations. They always had information regarding accommodation rates and transport fares to the different locations they moved to. They had information on who to first contact when they got to their destination and how to strategically identify clients in each location. When these sex workers got to the community, they went to bars and lodges as they would stay there for a short time before they moved again. However, if the sex workers came into an area and found that business was not as good as they had anticipated or if their marketability was reduced after staying in the community for some days, they would make phone calls to friends in other locations before they moved off again. The period these sex workers stayed in an area varied from one day to a couple of days or even weeks depending on the prevailing earning opportunities. Sometimes women would fail to get better prospects in the places they moved to and moved quickly to another location on the basis of information provided within their network.

The sex workers especially, they connect in such a way that they easily network if one finds an opportunity existing somewhere and also enrol new people into the sex work network. The young women look at those who have been sex workers and envy them. Some of them have made it and they lead a good life, have property, and the rest. So, due to lack of employment, they are talked into trying out in the trade. Then they form a group. The new recruit, if she gets money the easy way recruits others into the same. That’s why in every group you find there is a senior person, and these groups move together into an area and follow the same direction. They are connected by the same goal; contacts are by phone and social media. And through friends that they make, they keep their trade on track. To be connected, these people make sure they make as many friends as possible in the areas they operate from and keep in touch (woman, trader, 38 years, UG1).

Women in sex work ensured they maintained a chain of friendships and connections with the different information sources some of whom were men. These other information sources included lodge owners/ operators and the boat crew, who regularly travelled and always had current information on fish availability/abundance within the different fish sites.

When you talk to them, because as we always lift them to the boats, some of them have become our friends. They even ask you whether it's ok where they are heading to, and as we work here, we are aware of the situation happening in the islands through the traders, the boat skippers and other fishermen. When it is good, some of them [women] even tip us on their way back if all went well (man, 46 years, key informant-leader, boat loaders crew, UG1).

Besides networking with fellow sex workers, they also networked with fishermen, their biggest clients. Fishermen were reported to telephone inviting sex workers to follow them to the different islands/fishing communities that they moved to in the course of their work. Even other women in service provision like restaurant/bar operators, laundry and shopkeepers (to a small extent) also networked with fishermen and moved following the fishermen, especially when the latter moved for longer periods of up to 3 months or more.

Like these fishermen who go to look for Nile perch, they stay away for a long time, we hear that they could ask women like sex workers to find them in certain islands where they dock to buy more fish especially if they have home-made ice containers on their boats. (man, key informant-local leader, UG1)

You find that sex workers also move to the different islands and their sole purpose is to find places where they can do sex work. And they are usually alerted by their friends doing the same work, or the lodge owners” (…). “In the case of sex workers and fishermen, mobility to where business is good is generated by phone calls from fellow workers (woman, 57 years, key informant –VHT, UG2).

##### Hotel/restaurant workers’ networks

In Tanzania, women working at eating places (small hotels/restaurants) also engaged in transactional sex. They had their own network whereby they formed groups of 5–10 women who closely worked together. Their closer association helped them to locate places of high fish catches. They would meet together and allocate scouts to survey the business environment, once they identified good opportunities, they would move to the area.

Hotel workers move together as a group to places with high fish catches. The areas with high fish yield have large collections of people creating high demand for food and sex. The size of the group moving to a particular island or landing site depends on the population of the place of destination and the number of small hotels available in the area (woman, 39 years, food vendor, TZ).

##### Women fish traders/processors and fishermen networks

Other social connections were among women traders including fish traders/processors and the fishermen or their fish suppliers. Women in fish processing/trading were reported to be very mobile and these women moved with or followed the fishing crew. These could take up to three months before moving back to communities they considered as home. Being away from their homes for long periods of time exposed the women to multiple sexual partners and marriage/relationship break ups. For example, women engaged in fish trading/drying/smoking tended to move from one fishing community to another following the fishermen to secure fish or following their employers to process the catch, including drying fish. Their network helped them with security among themselves from men who mistreated them or from their employers and also simplified access to information about work opportunities including availability of, and markets for fish.

Fish processors, are connected in such a way that if one finds an opportunity existing somewhere they communicate and invite one another (woman, fish processor, 45 years, TZ).

In their quest for fish, women fish processors/ traders reportedly engaged in unprotected sex with fishermen to secure fish at a good price. Given that they moved from one fishing community to another, women sometimes had multiple sexual partners at the different fishing communities, which exposed them to sexually transmitted infections including HIV. In addition, this exposed them to sexual violence. Fishermen were reported to deny women fish if a woman declined providing sex, which left the women with a choice to make; to either provide sex and get fish cheaply or buy elsewhere at a higher price.

You may find that a woman gets fish from several men and she is mixing them all up. Sometimes when men realise that the woman is getting fish from several men, they sometimes gang up and beat her (woman, fish trader, 30 years, TZ).

##### Bar owners’/worker’s networks

Bar workers reportedly invited peers from mainland villages nearby for work in fishing communities. To a lesser extent, there were also middlemen on the mainland who recruited young girls for the bar owners because the latter changed employees frequently as a way of attracting bar customers.

… at all those places I have mentioned, I have brokers who call me and inform me of girls who are looking for what to do. So I tell them to send the girls and they come. So every time, I need a girl to work, I call those people (man 44 years, bar owner, UG1).

On coming to the fishing communities, most of the girls did not plan to work in bars but on reaching these communities, bar work would be the easiest and readily available option. In most cases, such girls were not paid by the employers but earned through providing sex to the bar customers. Similarly, bar owners at different fishing communities had a relationship whereby they exchanged or swapped bar workers. In such instances, girls/women who were less independent or could not easily find work would have no option but to move to where the next employer would take them.

This kind of work exposed the girls/women to the risk of HIV, not only through provision of sex to the bar customers but also through increased mobility since they kept changing places of work.

They (the bar owners) keep changing these girls and bringing in new ones, because girls especially new ones attract clients to come to bars. So when bar clients get tired of seeing these old faces, they (bar owners) change and bring in new ones (woman, 19 years, waitress, UG2).

##### Women: family networks

These networks included fishermen and their wives/long-term partners as well as women and their families. Women moved to follow their fishermen husbands/partners. Apart from a few women who moved and permanently stayed with partners at the fishing community, this type of mobility was in most cases short-term. Women moved for a day, two days but did not go beyond one week. For example, a fisherman could ask his wife to go and find him where he went fishing. Besides, women could be travelling on behalf of the husband who could be away fishing to attend to the husband’s wider family affairs like burial ceremonies or visits to relatives. Other women had left their families, including children and partners on the mainland and thus made frequent or regular trips to attend to their families. This was because raising a child in a fishing community was reported to expose the child to HIV risk at a young age and so some parents preferred to leave their children in a rural home with relatives or to keep them in boarding schools.

Much as there was this connection between women and their families, some women especially those involved in sex work never wanted their families to know the kind of work they were involved in. So, some ended up changing their names, concealing their whereabouts as well as their identity, which resulted in a weakening of family.

#### Benefits from belonging to a network

Participants reported benefiting from the social groupings, through information sharing, group protection especially from violence and financial support. For instance, network members not only offered support by informing peers about prevailing business/income prospects but also tended to move in groups to offer support to each other. For example, different groups of sex workers were reported to sometimes get involved in fights over sexual clients causing physical/bodily injury. For this reason, they preferred to move in specific groups as a form of protection. Others that commonly moved in groups were the bar/restaurant/hotel workers, who also moved together to provide support to each other.

Hotel workers travel in a group for purposes of protection. They are in the same surroundings to protect against crime and persecution, and to help one another in the case that one of them is faced with a problem (woman, 39, food vendor, TZ).

Some of the women move in groups for purposes of security, most especially when they are going to places they are not used to. Here even when they have arrived at those places, they want to stay near one another for protection in case one of them is forced into having or doing something not accepted by the others. For example, some men force themselves onto these women, and this person (the offender) can be dealt with in a group, even when the others are still serving their customers, they are usually forced to abandon whatever they are doing in order to protect their fellow sex worker (man, 46 years, boat loader, key informant, UG1).

The youthful sex workers are often seen to move in groups of three to five, and they have a linkage that they are either coming from the same place or have met on several occasions and have a tight connection. When planning to move to the islands, they meet somewhere to move together (woman, 39 years, key informant, restaurant owner, UG2)

Traders including fish traders were reported to also move in groups in case they are moving to the same market. In this way, they would load their goods on one boat and were assured of group protection and bargaining power to agree and set a good and uniform price for their goods.

Overall, women considered these groups as being important, especially in terms of group support and protection. They reported violence had occurred in the past, including the death of fellow women who did not belong to a group.

Some time back, a sex worker was murdered by her customer, and she had come alone here in XX to work. Another sex worker had her private parts mutilated by a customer who used a razor blade to cut them and run away. She almost died because a lot of blood was coming out of her (woman, 38 years, trader, UG1).

#### Risks from group membership

In addition to benefits, there were also risks from being a part of a network, which included exposure to HIV acquisition, violence and family breakages. Some younger women were reported to move because of peer pressure in a group and they find themselves living on the islands for good. Some of the women did not easily find shelter, so they stayed with friends or hired lodges for some time as they looked for a place to stay. Some of these places were prone to thieves, flooding during rainy seasons, and were poorly constructed. This was a particular problem for groups like bar workers who moved often and stayed in poor accommodation because they were working in a place for a short time.

Some of the peer groups lead women into risky behaviours, for example, many women are connected by their peers to men, but not knowing the outcomes of those connections. Many of them have been harassed by the men they were connected to by their friends. This usually happens to bar attendants (female, 31 years, bar worker, UG2).

Sex workers were greatly exposed to a number of risks including HIV risk, some of which resulted from their mobility:

…In addition to facing violence, they (sex workers) contract diseases and keep on spreading them. She gets it from here and takes it (the disease) to the islands and then from there to XX town (woman, 43 years, Bar owner, UG1).

Women fish traders reportedly made losses, especially during the rainy season and this usually exposed them to the risk of acquiring HIV:

Silver fish [a small type of fish that is dried in the sun] traders face the highest risk as they make big losses during the rainy season. Their silver fish could be washed away or it could rot, which could even result in involvement in sex for fish (woman, fish processor, 46 years, UG1).

Mobility related risks not only affected those who moved but also their families as long-term mobility reportedly led women to abandon their children, which not only affected the children but the communities as well. Other women were reported to move with their children which exposed the latter to the hardships that came along with the moving.

## Discussion

We set out to understand the different social networks and their interaction with women’s mobility patterns and HIV risk among women working in fishing communities of Lake Victoria. We found various social groupings that influenced the mobility among women in these areas. We found that most of the movement among the women was work related and was triggered by communication within the networks, mainly aided by phone calls, phone text messages and social media (especially WhatsApp).

Our results corroborate findings from other studies which show that most of the movement by the women in fishing communities is work related and often followed fish catches [27, 34]. Whereas earlier studies found that fishing activities followed fish migration patterns, causing women to follow the fish and the men, our study found that women’s social ties also played a significant role in influencing their mobility patterns.

Apart from fuelling women’s mobility, the networks included an aspect of social support to network members especially in the execution of their work. Studies have further shown that social networks are key in providing guidance on negotiating local circumstances including providing information on how or where to access sexual partners but with limited discussion on safe sex in these contexts [35]. We had similar findings in our study whereby networks mainly focused on ways of maximizing earning prospects and protection from harm, especially from violent clients and rarely focused on ways of avoiding HIV or other sexually transmitted infections (STIs). Although to a lesser extent, there were reports of protection by peers against men who wanted to force women into unprotected sex, which was likely to expose women to HIV risk, it is not clear to what extent HIV prevention was emphasized within the networks. Other researchers have suggested that networks could also serve as channels for effective HIV prevention programs [35–38]. Social networks have been shown to be effective in influencing the views and actions of peers in terms of peer education and support [39, 40]. These approaches, utilizing social networks, have the potential to increase HIV-related social support and thus improve HIV-related outcomes.

The appropriate utilization of the networks, especially through network members/leaders who clearly understand the mobility patterns and dynamics of the network members can help improve retention in research and, importantly, in health intervention programmes.

In reference to theories on social capital (whereby networks/relationships gained can be used to effectively achieve common goals), social ties and relationships may possibly produce a preventive effect against potentially harmful behaviour [41], such as reduced retention in health care, therefore they could be useful in the promotion of health interventions. Where mobility has been associated with interrupting access to health care and increasing risk among mobile individuals, the threat of losing social bonds can be powerful in inhibiting this potentially harmful behaviour. If the positive functions of social networks are clearly embedded within health interventions, especially the new models of differentiated care or differentiated service delivery of patient/participant-led adherence groups [42], networks could be used as channels for interventions on HIV prevention. For some studies, the influence and role of social networks on the behaviour of network members influenced the construction of social norms about sexual behaviour, challenging and supporting beliefs, assessment of risk and encouraging or discouraging HIV and STI testing [43, 44]. Explicitly, the different social networks, as seen in our findings, could be tapped, to help promote health awareness within the groupings/networks especially for key populations, including these mobile women in fishing communities.

While some research has shown the importance of social networks in providing support, mostly material and economic within and among network members [35], our research showed both negative and positive elements in the networks. In research in Zambia, peer support and social influence have been found to be important in protecting against HIV and other STIs [45]. The positive elements, including information sharing and group support observed in networks among these key populations could be used to advance health interventions. For this, there is need for a deeper understanding of the interlinking of people as well as the different forms of support within these fishing community networks in successfully utilising them to promote health information and behaviours.

Although the study focused on women in fishing communities, some of these women were sex workers who operated under diverse contexts not limited to fishing communities. This is a strength to the study as our findings may apply to mobile women in other contexts. Another strength was the application of multiple methods for data collection, namely key informant interviews, interviews with the women themselves, and observation of daily life in the fishing villages over a period of two months, which enabled the triangulation of the data from the different sources, thus strengthening and giving more validity to our findings. In addition, the study was carried out in three countries, using uniform methods and close collaboration within the team to understand common features. A limitation was the focus on HIV, which although widespread among women in these communities, is not their primary concern. However, our data did not capture the broader women’s sexual and reproductive health and rights contextual factors, including details of their own focus on maximizing earning prospects and protection from harm. A further limitation to our study was the absence of women whose lives were being studied on our research team, given the growing recognition of the value and importance of co-production of research or inclusion of peer researchers on research teams. Also, there was a risk or possibility of losing important information in case a researcher did not accurately take notes during the interview.

## Conclusion

Women resident and or working in fishing communities of Lake Victoria in East Africa had different social groupings created to facilitate the execution of their work. The networks played a big role in shaping and determining women’s mobility patterns within the different geographical locations, leading to extended sexual networks and potentially increasing HIV risk. Whereas these networks tended to increase risk, they also offered different forms of support, including information sharing, financial and social support to the network members, an indicator of the divergent positive and negative roles of the networks. The different social groupings within and among mobile women in fishing communities of Lake Victoria could be utilized to tailor effective HIV prevention and care interventions.

## Supplementary Information

**Additional file 1**. Social networks thematic coding matrix.

## Data Availability

The dataset generated and/or analysed during the current study is included in this article.

## Abbreviations

TZ: Tanzania
UG: Uganda
VHT: Village Health Team
STI: Sexually Transmitted Infection
AIDS: Acquired Immunodeficiency Syndrome
CAB: Community Advisory Board
UVRI REC: Uganda Virus Research Institute Research Ethics Committee
UNCST: Uganda National Council for Science and Technology
SERU: Science and Ethics Review Unit
NatREC: National Health Research Ethical Committee
MRC/UVRI and LSHTM: Medical Research Council/Uganda Virus Research Institute and London School of Hygiene and Tropical Medicine
MITU: Mwanza Intervention Trials Unit
RCTP: Research Care and Training Program
KEMRI: Kenya Medical Research Institute
UVRI-IAVI: Uganda Virus Research Institute-International AIDS Vaccine Initiative
LSHTM: London School of Hygiene and Tropical Medicine
IAVI: International AIDS Vaccine Initiative
LVCHR: Lake Victoria Consortium for Health Research
